# The unpredictable carbon nanotube biocorona and a functionalization method to prevent protein biofouling

**DOI:** 10.1186/s12951-021-00872-x

**Published:** 2021-05-05

**Authors:** Lorena García-Hevia, Mahsa Saramiforoshani, Jorge Monge, Nerea Iturrioz-Rodríguez, Esperanza Padín-González, Fernando González, Lorena González-Legarreta, Jesús González, Mónica L. Fanarraga

**Affiliations:** 1 The Nanomedicine Group, University of Cantabria-IDIVAL, 39011 Santander, Spain; 2 Osakidetza, Basque Center for Blood Transfusion and Human Tissues, Galdakao, Spain. Cell Therapy, Stem Cells and Tissues Group, Biocruces Bizkaia Health Research Institute, Barakaldo, Spain; 3 Department of Chemistry and Process & Resource Engineering, University of Cantabria, Barakaldo, Spain; 4 Present Address: Istituto Italiano Di Tecnologia, Smart Bio-Interfaces, Viale Rinaldo Piaggio 34, 56025 Pontedera, Italy; 5 Present Address: Department of Chemistry, Royal College of Surgeons in Ireland, Dublin, D02 YN77 Ireland

**Keywords:** Carbon, MWCNT, SWCNT, Serum, SDS-PAGE, Biotechnology

## Abstract

**Background:**

The intrinsic physicochemical properties of carbon nanotubes (CNTs) make them unique tools in nanotechnology. Their elemental composition, resilience, thermal properties, and surface reactivity make CNTs also of undisputed interest in biotechnology. In particular, their extraordinary ability to capture biomolecules on their surface makes them essential in this field. The proteins adsorbed on the CNTs create a biological coating that endows them the ability to interact with some cell receptors, penetrate membranes or interfere with cell biomechanics, thus behaving as an active bio-camouflage. But some of these proteins unfold, triggering an immune response that unpredictably changes the biological activity of CNTs. For this reason, the control of the biocorona is fundamental in the nanobiotechnology of CNTs.

**Results:**

Using TEM and AFM here we demonstrate a significant increase in CNTs diameter after protein functionalization. A quantitative analysis using TGA revealed that between 20 and 60% of the mass of functionalized nanotubes corresponds to protein, with single-walled CNTs capturing the highest amounts. To qualitatively/quantitatively characterize these biocoatings, we studied the biochemical "landscape" of the proteins captured by the different nanotubes after functionalization under various conditions. This study revealed a significant variability of the proteins in the corona as a function of the type of nanotube, the functionalization temperature, or the time after exposure to serum. Remarkably, the functionalization of a single type of CNT with sera from various human donors also resulted in different protein landscapes. Given the unpredictable assortment of proteins captured by the corona and the biological implications of this biocoating, we finally designed a method to genetically engineer and produce proteins to functionalize nanotubes in a controlled and customizable way.

**Conclusions:**

We demonstrate the high unpredictability of the spontaneous protein corona on CNTs and propose a versatile functionalization technique that prevents the binding of nonspecific proteins to the nanotube to improve the use of CNTs in biomedical applications. 
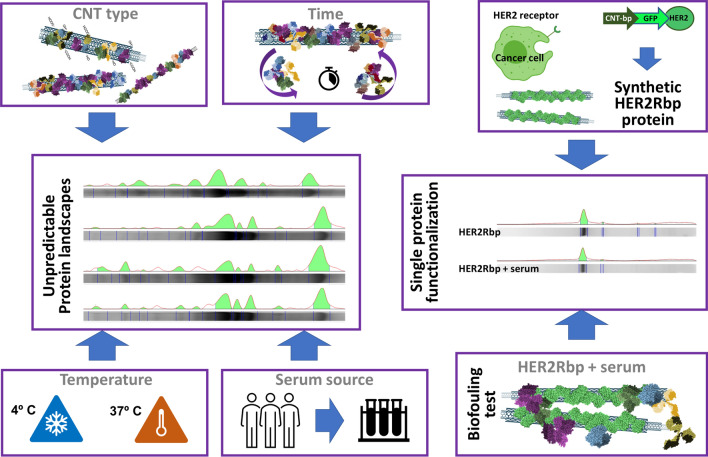

**Supplementary Information:**

The online version contains supplementary material available at 10.1186/s12951-021-00872-x.

## Background

Carbon nanotubes (CNTs) represent a highly versatile heterogeneous family of uni-dimensional nanomaterials that display many extraordinary properties in different industrial fields [1, 2]. These carbon-nanofilaments, discovered by Iijima in 1991 [3] have held a fundamental role in nanotechnology due to their outstanding physical–chemical behavior that makes CNTs indispensable for many industrial applications, being some of the most promising candidates for the design of multifunctional nanomaterials. More recently, CNTs have been incorporated into the biotechnological field driving major advances and a large potential impact on medical developments. Among the CNTs properties, stands out one with important implications in biotechnology, their high surface reactivity. These nanomaterials have a huge surface-to-weight ratio, calculated in ca. 1000 m^2^/g for single-walled CNTs (SWCNTs) and ca. 350 m^2^/g for multi-walled (MWCNTs) (5 walls) [4], a fact that, together with the intrinsic reactivity of graphene, makes the surface of the CNTs have an enormous capacity to interact with their environment and capture all kind of molecules from any source. This superficial property of CNTs that represents a breakthrough in the fields of drug delivery or nanotherapy, can also be a major handicap if not properly controlled. In this sense, CNTs can behave as Trojan's vectors introducing undesired chemicals or toxins in living organisms [5–7]. But this property makes CNTs excellent delivery vectors for transporting many different molecules, that do not need to be chemically modified for their loading. Among others, CNTs have been used to transport doxorubicin [8–10], or 5-fluorouracil [11–14], but also large quantity/variety of biomolecules including nucleic acids [15–17], or proteins [15].

When CNTs are exposed to biological environments, they get coated with the surrounding biomolecules, mainly proteins, which are active components that significantly modify the properties of nanotubes in biological contexts. Detailed proteomic identification studies have documented the promiscuous nature of CNTs where more than 750 different human proteins have been identified on the nanotube corona upon exposure to human serum or human cell extracts. Among these, immunoglobulins, albumin, fibrinogen, and complement are the most abundant [17–21]. The implications of the coating are pivotal as it provides CNTs with a bio-identity, transforming these nanomaterials into bioactive nanostructures that can participate or interfere in specific cellular functions, often triggering unpredictable interactions. For example, albumin on nanotubes triggers receptor-mediated endocytosis by most cells [22–25], while antibody or complement adsorption on their surfaces can trigger recognition by phagocytes, and elimination [26].

But CNTs, often taken as a unique nanomaterial, are morphologically and chemically diverse, and this is important in their interaction with the surrounding biomolecules. Previous studies show how the initial binding forces implicated in protein adsorption onto CNTs are hydrogen bonds [27]. Depending on the nanotube diameter and curvature [28], protein structural changes -such as partial unfolding- are also important in the final protein coating [19, 29]. This way, small nanotube diameters of ca. 1–2 nm—in the range size of globular proteins—have been reported to prompt more structural changes in proteins than tubes of smaller or larger diameters [19, 29]. Furthermore, recent studies show how the degree of folding of these corona proteins can significantly trigger inflammation, including the production of proinflammatory cytokines and innate immune responses [29]. All these facts suggest that spontaneous CNT coronas and their effects are highly unpredictable, and this could elicit completely different cellular and/or organic responses. In line with this, the diameter of the nanotube has been shown to trigger different biological effects [3, 19, 30–34]. SWCNTs, for example, preferentially cause DNA damage [35, 36], while MWCNTs mainly interfere with the cytoskeletal proteins, hindering cellular biomechanics [37, 38].

Hence, given the in vivo implications of CNTs corona, here we want to understand the factors that influence the protein coating of nanotubes. In this study, we take into account factors such as the properties of the surface of the nanotubes, the size of the nanotube, the temperature during functionalization, or the particularities of biological media, all critical aspects in nanobiotechnology. Finally, we develop a method to customize and control the CNTs corona with recombinant proteins designed and produced *ad-hoc*. This method also prevents nonspecific protein biofouling and denaturation of the synthetic coating protein.

## Results

**Fig. 1:**
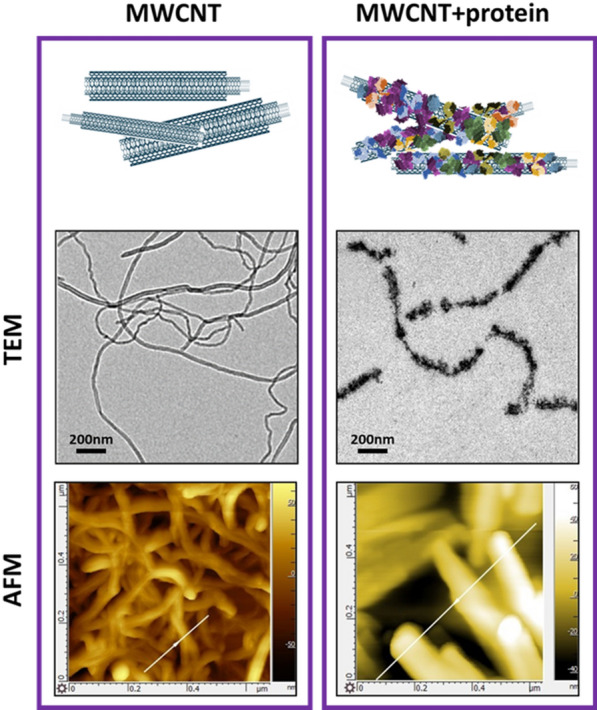
Functionalized MWCNTs significantly increase their diameter upon functionalization with proteins. TEM and AFM images of identical MWCNTs before (left) and after (right) serum functionalization. A significant enlargement of the nanotube diameter is observed upon protein coating. The uneven staining of the functionalized CNTs in the TEM images suggests a discontinuous protein coating on the nanosurface

Numerous studies show that CNTs capture a high number of proteins on their surfaces but few papers demonstrate the morphological and mass implications of functionalization. As a first objective, we wanted to identify morphological changes on CNTs upon protein coating using transmission electron microscopy (TEM) and atomic force microscopy (AFM). Figure 1 shows TEM and AFM images of pristine MWCNTs before and after functionalization with serum proteins. Both TEM and AFM techniques reveal a significant enlargement of the diameter of the tubes.

**Fig. 2:**
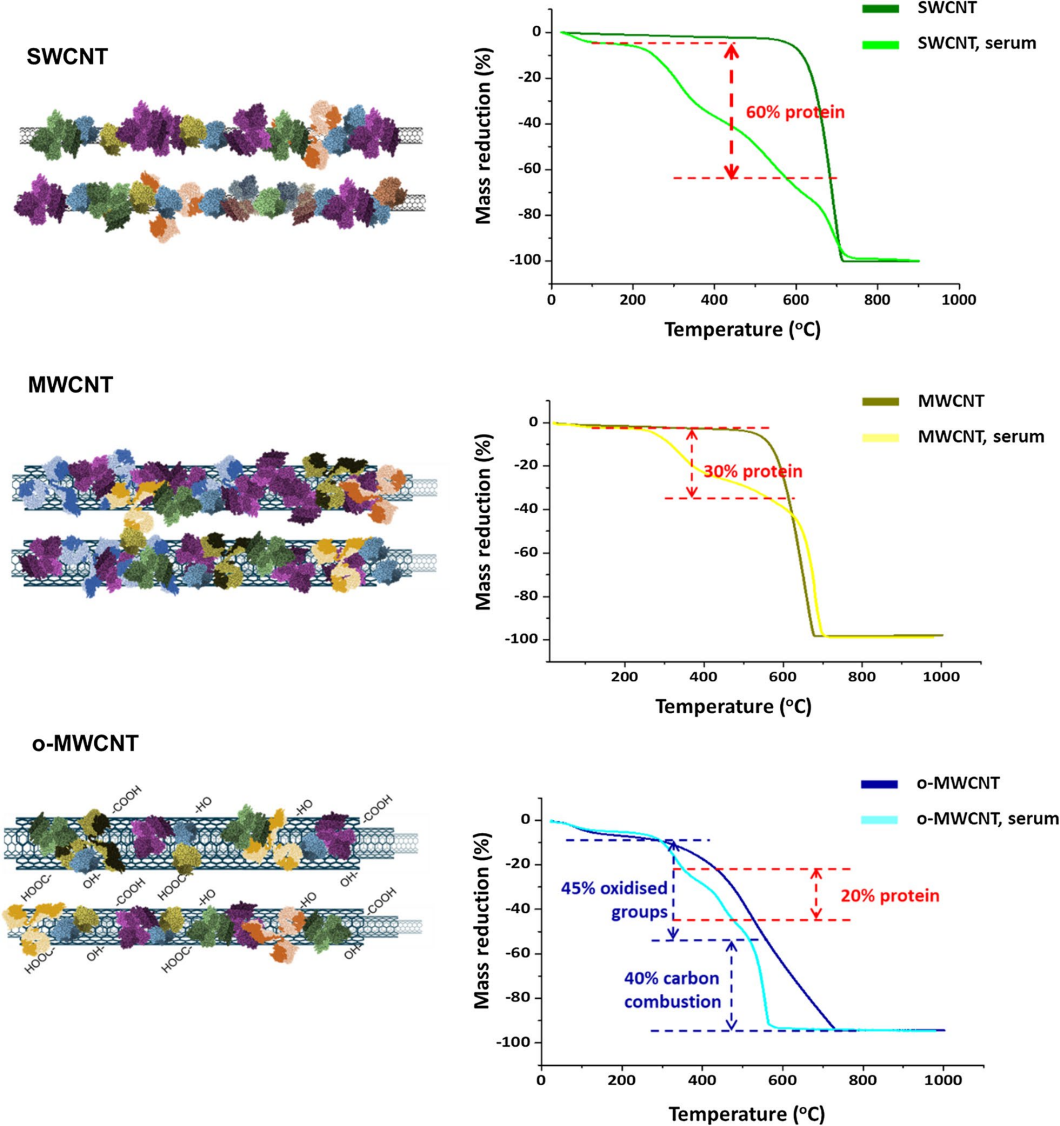
Thermogravimetric analysis (TGA) of SWCNTs, pristine MWCNTs, and o-MWCNTs. The calculated percentages of the final mass of the functionalized nanotubes corresponding to the biomolecular coatings are indicated in red

To calculate the amount of protein adsorbed on this bio-coating, we functionalized different nanotubes, namely pristine SWCNTs (SWCNTs), pristine MWCNTs (MWCNTs), and oxidized MWCNTs (o-MWCNTs) with bovine serum as a standard protein mixture (Materials and Methods), and performed a thermogravimetric analysis (TGA) in the presence of oxygen. Results shown in Fig. 2 demonstrate how, upon temperature rise, pristine (non-functionalized) SWCNTs displayed a single mass loss observed at approximately 600 ºC, corresponding to the combustion of carbon. In serum functionalized SWCNTs the loss of mass below 600 ºC corresponded to the combustion of organic matter. This mass reduction was observed in two steps, 200–400 ºC, and 400–600 ºC. Thus, the calculated loss of mass corresponding to biomolecules (mostly proteins) in functionalized SWCNTs corresponded to approximately 60% of the mass of the functionalized nanotubes.

Pristine MWCNTs displayed a single mass loss observed at ca. 550 ºC, corresponding to the combustion of carbon. The same nanotubes functionalized with serum displayed a two-step mass reduction (two different slopes), at 250–400 ºC and 400–550 ºC as a result of the combustion of the organic matter. Here, the calculated mass of the biocorona approximately corresponded to 30% of the functionalized MWCNTs weight.

Finally, the mass loss observed in non-functionalized o-MWCNTs occurred in two slopes. The first (100–550 ºC), corresponding to the oxidized groups (approximately 45%), and the second (from 550 ºC), corresponding to the combustion of carbon, starting at approximately the same temperature as in observed for pristine MWCNTs. This second mass loss corresponded to ca. 40% of the mass. Upon functionalization with proteins, o-MWCNTs displayed an extra mass drop between temperatures 250–550 ºC that represented the total sum of the organic matter (serum proteins) added to the oxidized group combustion. Taking into account that the loss of mass that corresponded to the oxidized groups was 45%, the final calculated protein mass in functionalized o-MWCNTs was ca. 20% of the total weight of the biofunctionalized o-MWCNTs. Summarizing, the percentage of mass that corresponded to the protein adsorbed on the surface of the CNTs depended on the size and properties of the nanotubes, and fluctuated between 20 and 60% of the total mass of the functionalized CNTs, with SWCNTs capturing the highest amounts. These percentages confirmed that CNTs adsorbed enormous amounts of proteins, especially SWCNT (as represented in Fig. 2 diagram) and that in vivo, this bio-camouflage will be pivotal in driving CNTs interactions with the different biological systems.

Our next step was to understand whether the quantitative and qualitative composition of the corona was predictable or constant. To do this, we explored changes as a function of the CNT type, for it is known that the variety of proteins on the nanotube surface is strongly influenced by the diameter, the hydrophobicity, or functional groups on the surface of the CNTs [28]. For instance, oxidized nanotubes bind more variety of proteins than pristine nanotubes, and proteins that bind SWCNTs have a low affinity for MWCNTs [39]. But we would like to compare the proteins that bind to different nanotubes exposed to the same biological media -in this case, bovine serum- using an identical functionalization protocol.

To visually and straightforwardly evaluate these biocoatings -quantitatively and qualitatively -, we compare the different biochemical protein “landscapes” on functionalized CNTs. To do this, the total protein on the surface of the different functionalized CNTs was stripped with Laemmli buffer. The obtained protein solution was then loaded on SDS-PAGE gels for biochemical analysis and, upon electrophoresis, gel staining (Additional file 1: Fig. S3), scanning, and digital analysis, the protein “landscapes” were calculated. Hence these green profiles display a visual qualitative and quantitative representation of the protein distribution for each type of CNTs (Materials and Methods).

**Fig. 3:**
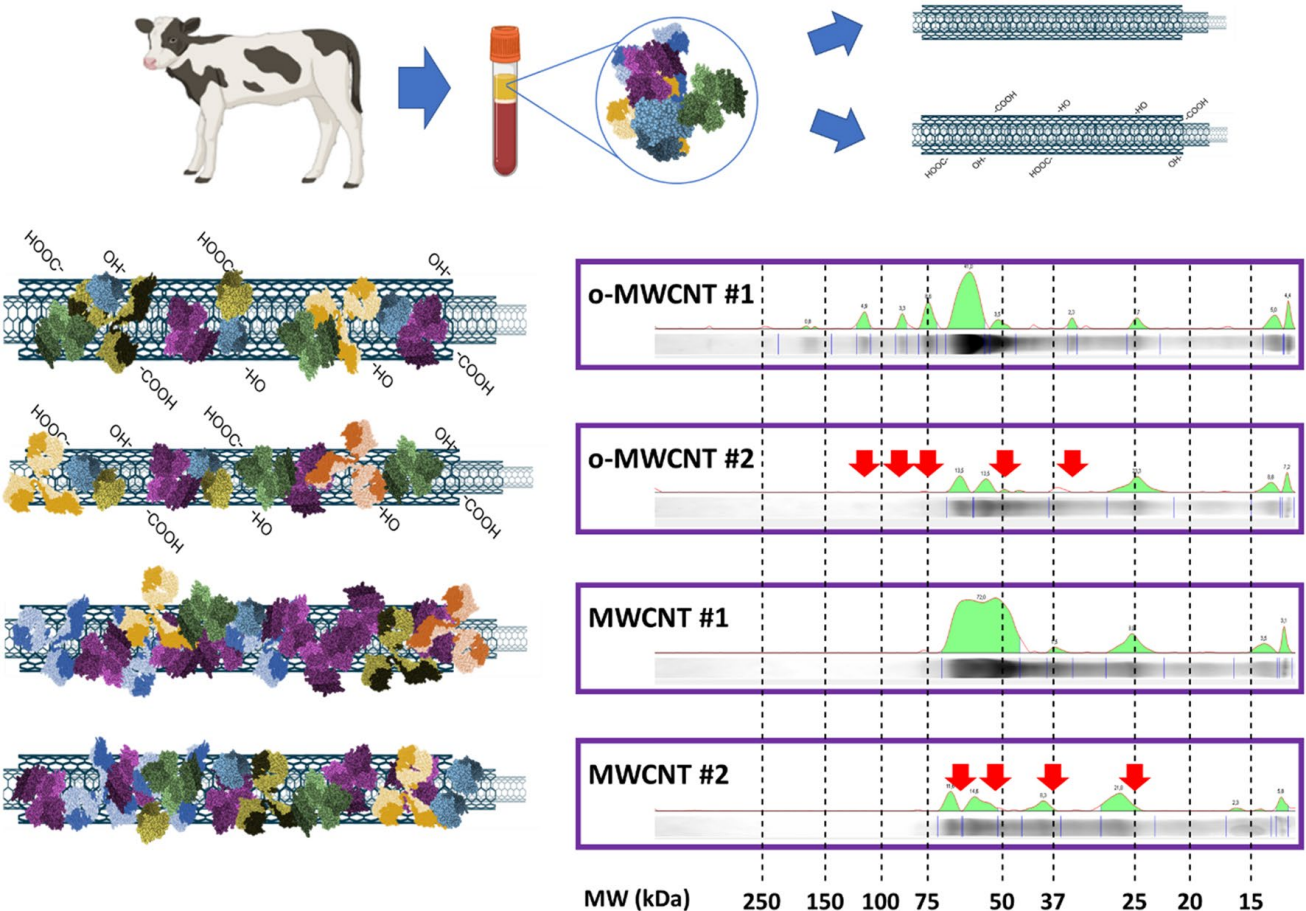
Biochemical landscapes of o-MWCNTs and MWCNTs functionalized with the same bovine serum. The qualitative and semi-quantitative protein landscapes (green profiles) were calculated from the SDS-PAGE protein analysis (in horizontal). These profiles demonstrate how identical protein components interact very differently with each nanotube. Arrows indicate some of the most divergent protein peaks. Molecular weights (MW) of the proteins are indicated at the bottom of the figure. The full SDS-PAGE analysis is shown in Additional file 1: Figure S3

As a first approach, we compared pristine and oxidized MWCNTs (o-MWCNTs). These two types of nanotubes often fall into one category in nanobiotechnology/nanomedicine despite they are considered different from the physicochemical point of view. For the study, we used two varieties of nanotubes of each type -two types of o-MWCNTs, and two of pristine MWCNTs. The nanotubes were functionalized in the same bovine serum following identical protocols (Material and Methods). Interestingly, upon biochemical analysis, we found unique protein “landscapes” for each nanotube type. Figure 3 shows how proteins that are visible in one of the o-MWCNTs types are not detected in the other oxidized nanotube (red arrows). Similarly, the two types of MWCNTs used in the study produced very different protein peaks in the landscapes (red arrows) which suggests that small changes on the surface of the nanotube could be very critical in the interaction with environmental proteins.

**Fig. 4:**
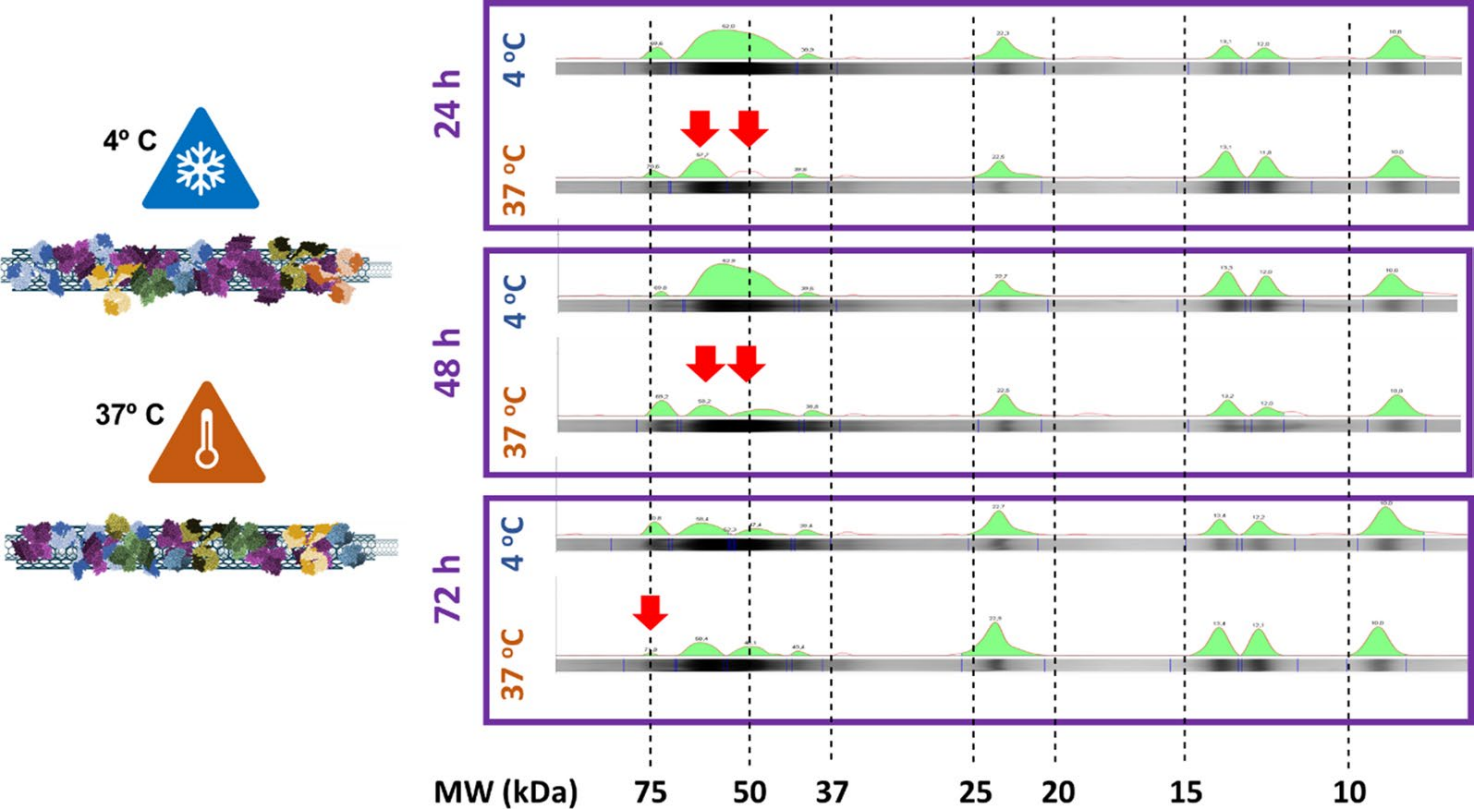
Biochemical landscapes upon MWCNTs incubation with serum at 4 ºC or 37 ºC. The protein landscapes obtained upon MWCNTs incubation with bovine serum at 4 ºC or 37 ºC during 24, 48, and 72 h. SDS-PAGE analysis reveals how the same protein components interact very differently with the same nanotube as a function of temperature and time. Arrows indicate some of the most divergent protein peaks. Molecular weights (MW) of the proteins are indicated at the bottom of the figure. The full SDS-PAGE analysis is shown in Additional file 1: Figure S4

Temperature is a key factor in protein folding and can significantly increase the flexibility and unfolding degree of polypeptides. Thus, the temperature at functionalization could also have important implications in nanomedicine since, depending on the preparation of the samples and their conservation, the in vivo behavior of CNTs could vary significantly [29]. Because of this, the next factor that we have analyzed was the influence of temperature on the interaction of proteins with CNTs as a function of time. To do this, we incubated a single representative type of (pristine) MWCNTs with a single type of serum at 37 °C and 4 °C for different times. As in the previous section, we examined changes in the protein landscapes on the surfaces of the nanotubes using SDS-PAGE analysis (Fig. 4, Additional file 1: Fig. S4). Figure 4 shows how nanotubes incubated at 37 ºC displayed fewer proteins in the range of 60–50 kDa on their surfaces compared to the same nanotubes incubated with the same serum but at 4 ºC during 24 and 48 h (red arrows). Again, these studies show how, for a single type of CNT, temperature and time conditions are critical factors in the final composition of the corona.

These findings suggest there could be small temperature-triggered conformational changes of the proteins that could cause changes in the composition of the corona, as observed in previous studies [19, 28]. From a biochemical point of view, physiological temperatures close to 37 °C allow proteins to be more dynamic and more flexible, and this could result in greater polypeptidic interaction with the surface of the nanotube. Most proteins, and serum albumin, in particular, exhibit great structural plasticity at physiological temperature and their polypeptides can spread over the surface of the CNTs, unfolding slightly, covering a larger surface of the nanotube, thus interfering with the binding of other proteins, more flexible or voluminous [29]. Interestingly, and as described for other types of nanomaterials [40], after 72 h of incubation, the originally adsorbed proteins were progressively replaced by others with different affinities for the nano-surface. However, these differences in the biocorona formed at 4 or 37 °C did not result in clear differences in the percentage of protein captured in the total mass of the functionalized nanotubes (see TGA in Additional file 1: Fig. S5).

**Fig. 5:**
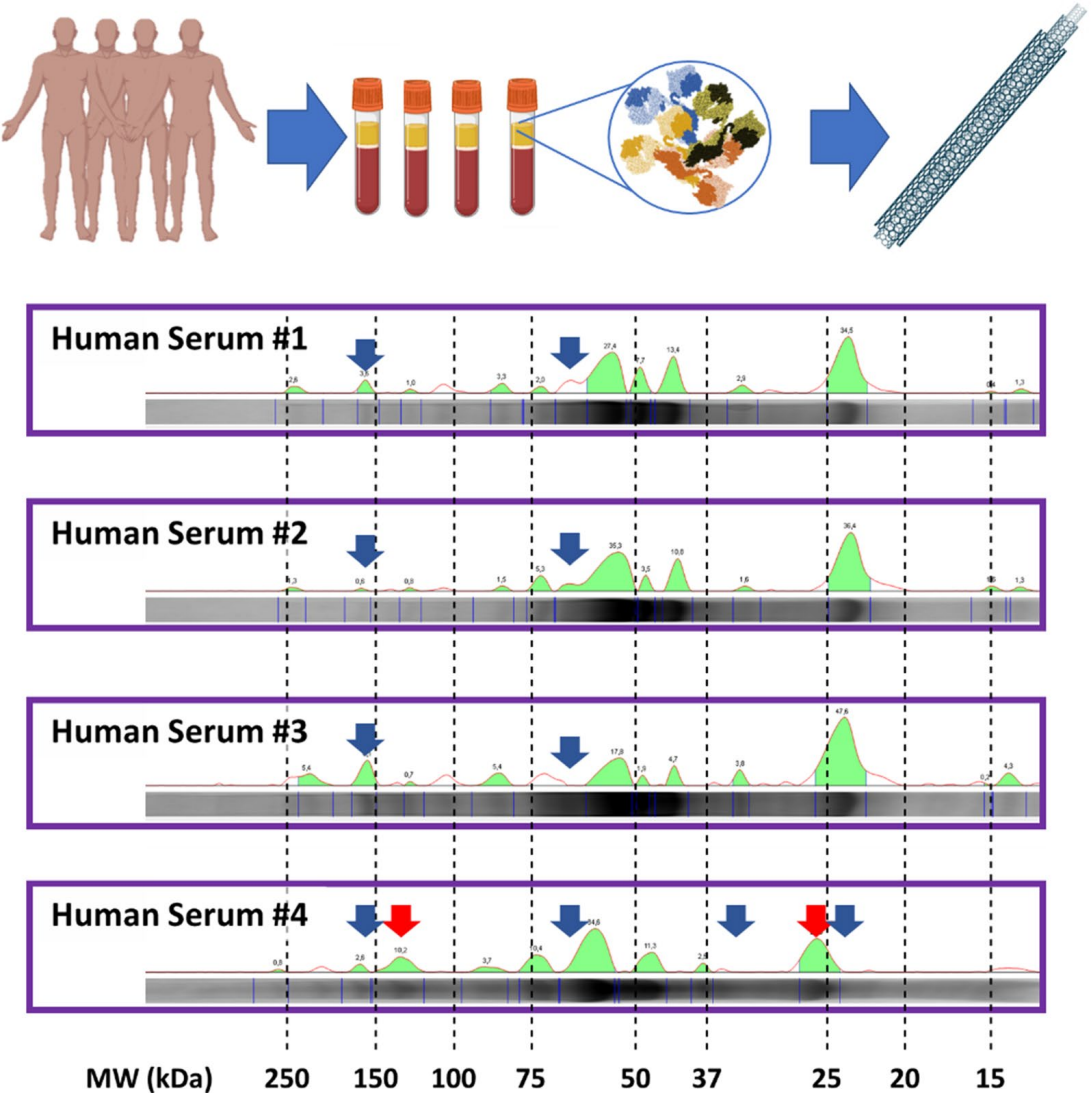
Biochemical landscapes of MWCNTs functionalized with 4 different human sera. Biocorona protein profiles were obtained upon incubation of a single type of MWCNTs with 4 different healthy human sera during 24 h. The biochemical landscapes reveal how the protein components of human sera interact differently with the same nanotube. Arrows indicate some of the most divergent protein peaks. Some proteins are completely absent in some of the samples (blue arrows) while others display significant changes in their affinity for the nanotube (red arrows). Molecular weights (MW) of the proteins are indicated at the bottom of the figure. The full SDS-PAGE analysis is shown in Additional file 1: Figure S6

Preceding literature details with high precision the great variety of proteins that bind to CNTs when exposed to human serum, plasma, or other biologically relevant fluids [17, 39, 41, 42]. Generally, studies agree that the most predominant proteins in these fluids, such as albumin, are present on the corona. However, each analysis lists a different list of proteins captured by the nanotubes. The different protein listings raise the question of what might happen if CNTs are exposed to sera from different human donors. Given our previous results, and to translate their significance in the context of nanomedicine, we now questioned if the same type of CNTs would interact with identical, or very similar, proteins upon exposure to different human sera. To investigate this issue, we exposed a single type of CNT to 4 different healthy human sera with known compositions (Additional file 1: Table S1). Interestingly, our results reveal that different sera interacted differently with the same type of CNTs. While some proteins do not appear on the surface of the nanotube and many changes their level of affinity (red/blue arrows respectively, Fig. 5). This means that the same type of nanotube could behave completely differently in each patient depending on the peculiarities and properties of their serum. Thus, this finding has important biomedical implications, as it suggests that CNTs -as well as any other graphene-coated nanosystems [43–49], could have completely different effects in different patients.

**Fig. 6:**
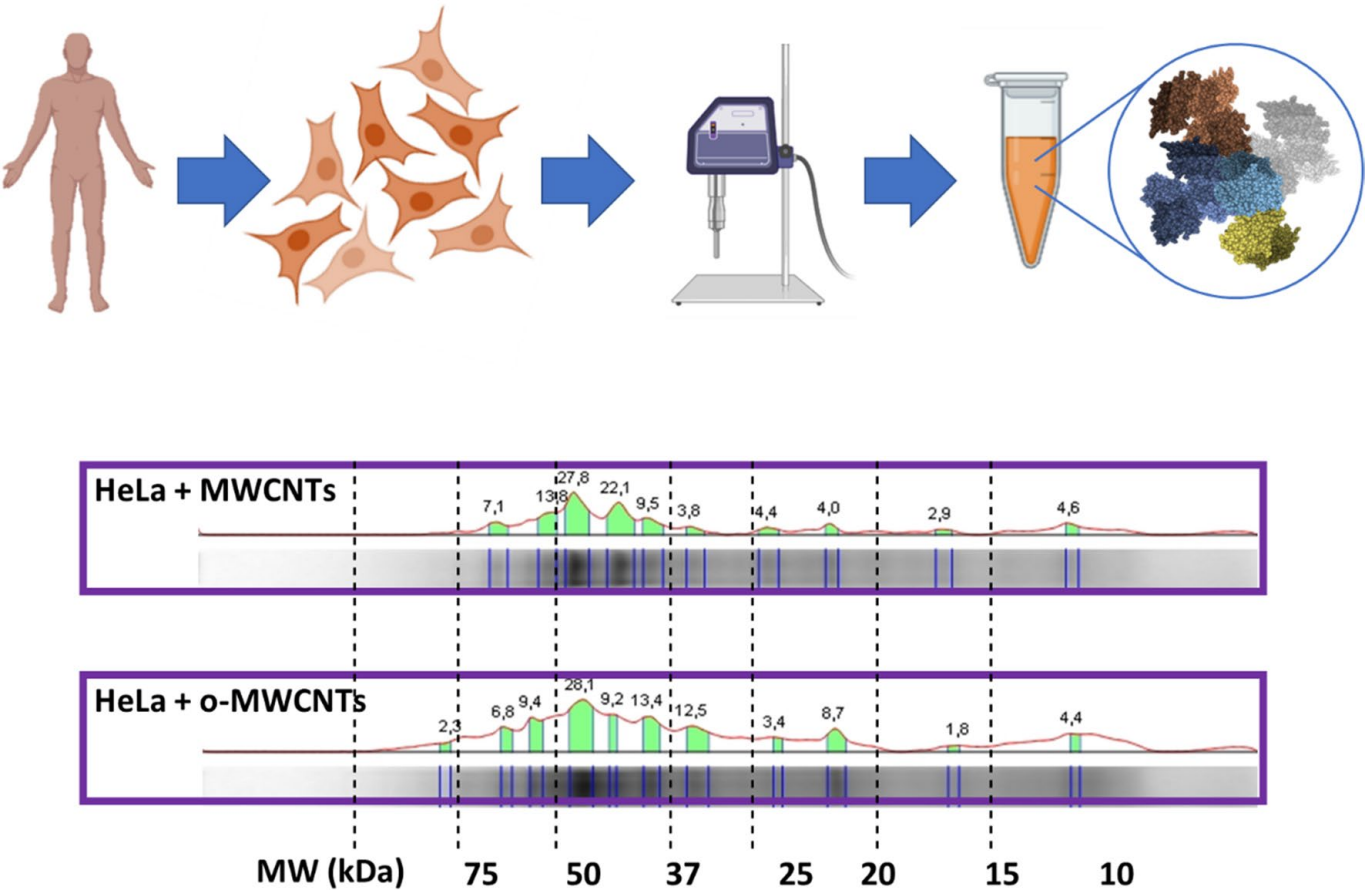
Biochemical landscapes of MWCNTs and o-MWCNTs functionalized with human intracellular proteins. The two types of nanotubes interact differently with the intracellular proteins obtained from HeLa cells. The full SDS-PAGE analysis including comparison with bovine and human serum proteins is shown in Additional file 1: Figure S7

Finally, to identify the protein landscape of the CNTs upon crossing the plasma membrane and binding intracellular proteins, we functionalized pristine MWCNTs and o-MWCNTs with a cell extract containing a mixture of intracellular proteins. To do this study, we chose human HeLa cells, a well-studied cervical carcinoma cell line in which CNTs have been shown to penetrate, triggering antiproliferative and pro-apoptotic effects [37]. On this occasion, the nanotubes were functionalized in a suspension of cytoplasmic intracellular proteins obtained from HeLa cell culture lysates. Figure 6 shows the distinctive protein profiles for the two types of nanotubes upon incubation with intracellular proteins. As expected, these protein patterns were also different from those observed when the nanotubes were functionalized with either bovine or human sera (Additional file 1: Fig. S7).

**Fig. 7:**
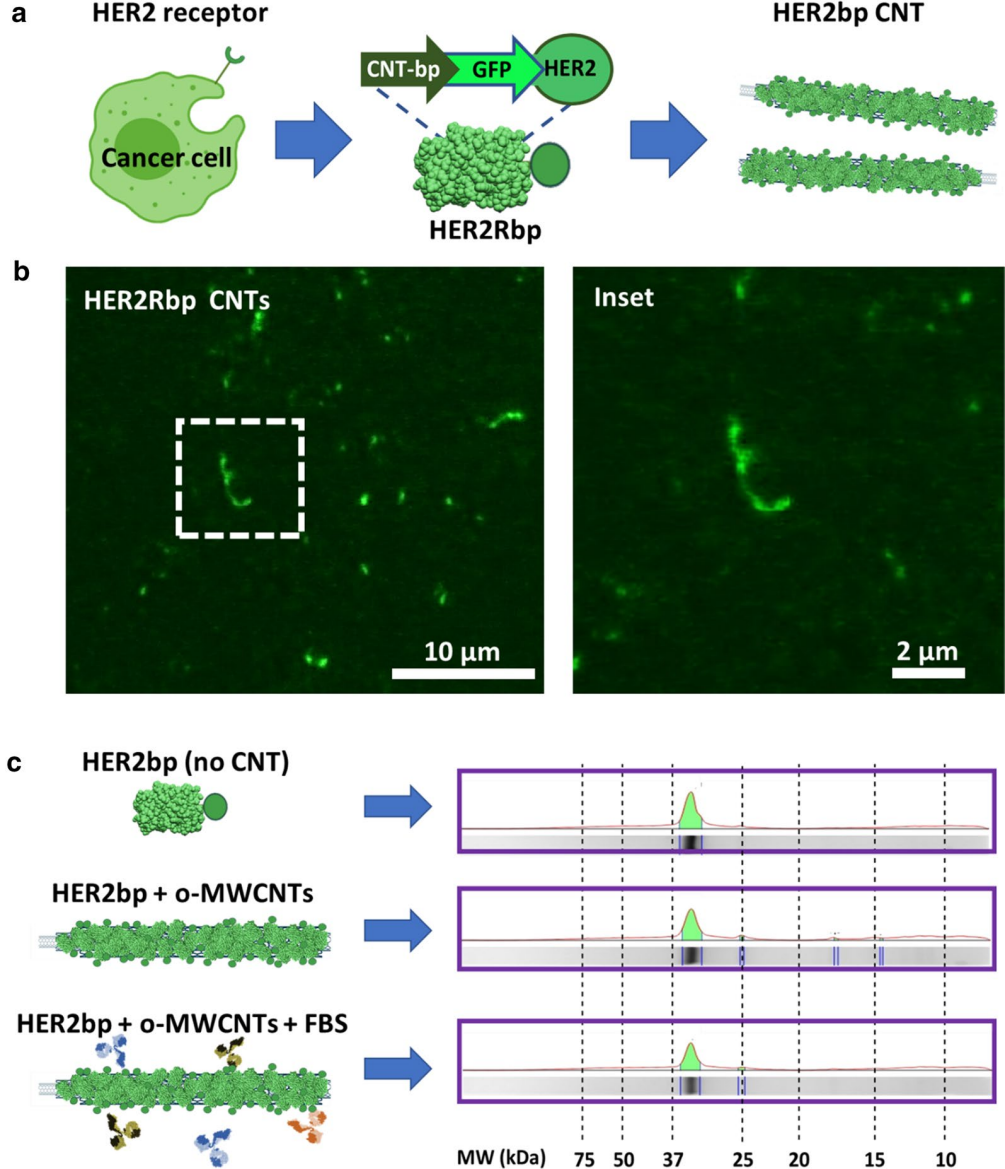
Genetically engineered protein design to prevent non-specific protein biofouling. **a** A recombinant protein design: CNT-binding peptide (CNT-bp) genetically attached to GFP and HER2 binding peptide. **b** Confocal microscopy image of fluorescent HER2bp-functionalized CNTs. Fluorescence indicates the GFP in the protein is not denatured upon attachment. **c** Protein landscapes of the purified HER2bp (top); the protein stripped from the HER2bp functionalized nanotubes (middle lane), and the total protein stripped from the functionalized nanotubes after incubation with serum. The presence of a single band demonstrates how the synthetic protein prevents unwanted biofouling of serum proteins on the nanotube

These results led us to design a predictable protein coating for nanotubes, to prevent non-specific interaction with the environmental proteins, a novel functionalization system that could also serve to improve CNTs targeting while preventing the activation of the immune responses and premature elimination of the nanotubes. For this purpose, we used genetic engineering to design a synthetic (recombinant) protein containing a peptide targeting the Human Epidermal Growth Factor Receptor 2 (HER2, also known as Neu or ErbB2). This receptor is overexpressed on the surface of multiple tumor cell types including breast [50, 51], ovarian [52], lung [53], stomach, oral cancers [54] and, its relatively low expression on normal tissues makes it a clinically useful molecular target for directed therapies. The synthetic protein sequence designed contained: (i) a cationic CNT-binding peptide [55] in the N-terminus, (ii) a fluorescent reporting protein (GFP), and (iii) a the HER2-binding peptide [56] at the C-terminus (Fig. 7a). The CNT-binding domain consisted of a 10xHis cationic peptide that can be attached to most proteins. This peptide is useful in protein affinity purification and prevents recombinant protein direct interaction with the nanosurface of the CNTs, thus preventing ligand–protein denaturation. In this case, denaturation would be detected by protein fluorescence quenching. The synthetic HER2-binding protein (HER2bp) was produced in bacteria and was purified using affinity chromatography (Materials and Methods). Functionalization of the nanotubes was performed with saturating amounts of the HER2bp recombinant protein resuspended in PBS (Materials and Methods). Functionalized CNTs became fluorescent (Fig. 7b), thus indicating the recombinant GFP-fused protein was correctly folded (native) upon binding to the nanotube. This result is interesting in the context of preventing the activation of the immune system resulting from the denaturalization (unfolding) of the CNT-interacting proteins [29].

To investigate if functionalization with this synthetic protein served to control the unwanted interaction of the nanotube surface with nonspecific proteins of the environment, we incubated HER2bp-functionalized CNTs with bovine serum for 16 h at 30 ºC. Figure 7c shows the landscapes of (i) the purified HER2bp protein, (ii) the protein stripped of the *as-prepared* HER2bp-CNTs, and (iii) that of the same HER2bp-CNTs after incubation with serum. The obtained biochemical landscapes demonstrate the efficiency of this functionalization method preventing protein denaturation and biofouling with unwanted environmental proteins (Additional file 1: Fig. S8). In summary, we demonstrate how the use of custom-made proteins, such as this HER2bp, can be a predictable way to functionalized CNTs for biomedical purposes.

## Discussion

The unpredictability of the biocorona composition makes it impossible to predict the fate and effects of CNTs in the in vivo context. Here we show how the final protein assortment on the nanotubes depends on many factors, namely (i) the surrounding media, (ii) the temperature, (iii) the exposure time, (iv) the type of nanotube -concerning its size and reactivity-, and, more interestingly, the individuality of the human sera. Since all these factors are critical for in vivo applications, we have developed a way to predictably control the nanotube surface with synthetic proteins created and produced ad hoc. We have designed a cationic peptide to stably bind synthetic (recombinant) proteins to oxidized nanotubes, and demonstrate that this attachment protects the CNTs from unwanted interactions with the environmental proteins. Since these custom-made CNT-binding proteins have a modular design, they can be designed *ad-hoc* according to the experimental needs simply by changing the targeting moieties, or fluorescent tags, to improve the predictability/localization of CNTs in most biological environments. Besides, this design can also be used to functionalized two-dimensional graphene-based nanomaterials to create unique nanostructures for biomedical applications. These important changes in the biocorona are a notable issue to consider in the in vivo context as the affinities and surface charge of these nanomaterials change. These modifications are can cause a change in targeting and, consequently, in the toxicity and possible cumulative effect of these carbonous nanomaterials [47, 48].

## Conclusions

Understanding and controlling the biomolecular coating on CNTs is the first step in nanobiotechnology and nanomedicine to be able to predict the biodistribution and effects of CNTs. This step is critical to producing reliable nanotherapies and nanovectors for in vivo applications. The protein corona endows CNTs with a biological identity that enables their intermingling with local components and participation in exquisite complementary molecular interactions [57]. These proteins determine the interaction of nanotubes with biological components, such as receptors, membranes, cytoskeletal filaments, etc., so understanding their variation and controlling their nature is essential in in vivo contexts. For this reason, we have developed a genetic engineering method to produce a stable custom-designed biocorona on nanotubes that allows predetermining the nature of the CNTs, and thus improve the biodistribution and effects of CNTs and CNT-based nanostructures.

## Materials and methods

### CNTs

Pristine MWCNTs (NC3100™) and oxidized MWCNTs (o-MWCNTs) (NC3101™) were purchased from Nanocyl. Pristine SWCNTs (900,711) and MWCNTs (698,849) (where indicated) from Sigma-Aldrich as powder. A characterization table is also provided (Additional file 1: Table S2). In-house o-MWCNTs were prepared as previously described [58, 59] using pristine MWCNTs (Nanocyl NC3100™) that were oxidized by sonication of a mixture of H_2_SO_4_/HNO_3_ (3:1) at 37 °C for 9 h under continuous shaking. o-MWCNTs were washed with a NaOH aqueous solution by three centrifugation/redispersion cycles (13,000 rpm). The acid solution was removed by filtration through a 0.45 μm Teflon membrane and was washed in H_2_O until pH ∼ 7.

### CNTs characterization

CNTs were characterized by thermogravimetric analysis, mass spectrometry, and FTIR in previous studies [60]. The unpolarized Raman spectra in Additional file 1*:* Fig. S1, S2 were taken with a Horiba T64000 triple spectrometer in the backscattering geometry, using the 514.5 nm line of a Coherent Innova Spectrum 70C Ar + Kr + laser and a nitrogen-cooled CCD (Jobin–Yvon Symphony) with a confocal microscope for detection. The laser beam was focused down to 1 μm spot with a 100 × objective and kept the power on the sample below 2mW to avoid laser-heating effects on the probed material and the concomitant softening of the observed Raman peaks (Additional file 1*:* Fig. S1).

### Serum functionalization

Functionalization of different CNTs was performed by nanomaterial dispersing in 30% serum protein solutions. CNTs were incubated at 37 ºC for 24 h unless otherwise indicated in the text. CNTs dispersions were washed by centrifugation for 2 min at 10,000*g*. Their concentration was determined by optical absorption at 550 nm using a calibration curve as previously described [61, 62]. Bovine serum (BS) was obtained from Gibco-Life Technologies (Cat No. 10108165). Human sera were collected from different donors at the Basque Center for Transfusion and Human Tissues (Additional file 1: Table S1). Sera were obtained by repeated cycles of vortex mixing followed by mild sonication (cycles of 5″ on, 5″ off, 10 min, frequency of 38 kHz).

### HeLa protein lysate preparation and functionalization

HeLa cells (ATCC® CCL-2) were cultured under standard conditions in Minimum Essential Medium containing 10% serum, and ampicillin (1:1000). Approximately 10^7^ cells were harvested by trypsinization, washed with phosphate-buffered saline (PBS), and incubated in lysis buffer. The cells were broken by pipetting and the supernatant was separated by centrifugation (13,000 rpm, 5 min). HeLa cell-lysate supernatant proteins were mixed with 200 μg o-MWCNTs were sonicated (5 min, 2 s pulses, 65% amplitude, 4 °C), and were washed 3 times with PBS to remove the excess protein.

### TEM, AFM, TGA

TEM images were obtained on JEOL JEM-2100 operating at 80 kV. Samples for TEM were prepared using ethanol as a dispersant and a drop of this suspension was adsorbed onto a lacey copper grid. Protein on CNTs was stained with lead citrate and uranyl acetate. AFM was performed using a WITec alpha-300 microscope on nanotube samples dried on a borosilicate glass coverslip. TGA was carried out using a Setaram Setsys Evolution 1700 apparatus, ranging from ca. 20–1000 °C. Measurements were performed under an air atmosphere with a heating rate of 10 °C min^−1^ at a total flow rate of 20 mL min^−1^.

### SDS–PAGE protein analysis

SDS-PAGE was performed on CNTs incubated with the protein solutions after repeated washed by centrifugation. All samples analyzed contained 30 µgr of functionalized CNTs. The nanotube concentration was determined by optical absorption as described previously [61]. Total protein on functionalized nanotubes was stripped with buffer Laemmli (0.125 M Tris–HCl pH 6.8, 20% glycerol, 4% SDS, 2% mercaptoethanol, 0.02% bromophenol blue) at 95 ºC for 10 min. The protein mixture was loaded on Mini-Protean® precast gels (BioRad) for SDS-PAGE analysis. The protein landscape analysis was obtained using Coomassie blue-stained gels that were scanned with a BioRad GelDoc EZ system. Protein patterning corresponds to Bio-Rad molecular weight standards.

### Gene synthesis, protein expression, purification, and CNT functionalization

The recombinant gene was synthesized (General Biosystems) and was cloned in pET 15b plasmid systems (Novagen). One Shot™ BL21(DE3) *E. coli* (Thermo Fisher) cells were transformed with the expression vector. The clone was cultured in LB broth medium, and when the optical density was 0.5 (590 nm) then Isopropylb-D thiogalactopyranoside (1.0 mM IPTG, PanReac AppliChem) was added. After 6 h at room temperature incubation, cells were collected by centrifugation and were resuspended in LEW buffer (50 mM NaH_2_PO_4_ and 300 mM NaCl in distilled water pH = 8.0). 1 mg/ml lysozyme and protease inhibitor (Pierce, Thermo Fischer) were added to lyses bacterial cells. Bacterial cell lysates were obtained by probe sonication (5 × 15 s pulses at 130 W, 65% amplitude, with 15 s intervals, 4 ºC) and insoluble material synthesized by centrifugation. Bacterial soluble protein lysate was loaded onto pre-equilibrated Ni-TED columns (Protino® Ni-TED, Macherey–Nagel GmbH & Co., Düren, Germany). Recombinant His-tagged protein was eluted in buffer supplemented with 250 mM imidazole. PD-10 Desalting Columns (GE Healthcare, Chicago, USA) were used to remove the imidazole and exchange buffer to PBS. Finally, saturating amounts of the recombinant protein were mixed with 200 μg o-MWCNTs, were sonicated (5 min, 2 s pulses, 65% amplitude, 4 °C), and were washed 3 times with PBS to remove the excess protein.

### Confocal CNT imaging

Functionalized CNTs were placed on a coverslip and imaged using a Nikon A1R confocal microscope (100 × 1.46 n.a. lens).

### Illustrations

Illustrations have been created with the BioRender software available at BioRender.com.

## Supplementary Information

**Additional file 1.** Additional figures and tables.

## Data Availability

All data and materials are included in this published article and its additional files.
