# Supplementary material for: The unpredictable carbon nanotube biocorona and a functionalization method to prevent protein biofouling
Source: J Nanobiotechnology. 2021 May 5;19:129. doi: 10.1186/s12951-021-00872-x (PMC8097984; doi:10.1186/s12951-021-00872-x)
Supplement: Supplementary file 1 — Additional file 1. Additional figures and tables. [file 12951_2021_872_MOESM1_ESM.docx]

# Supplementary Materials:

#
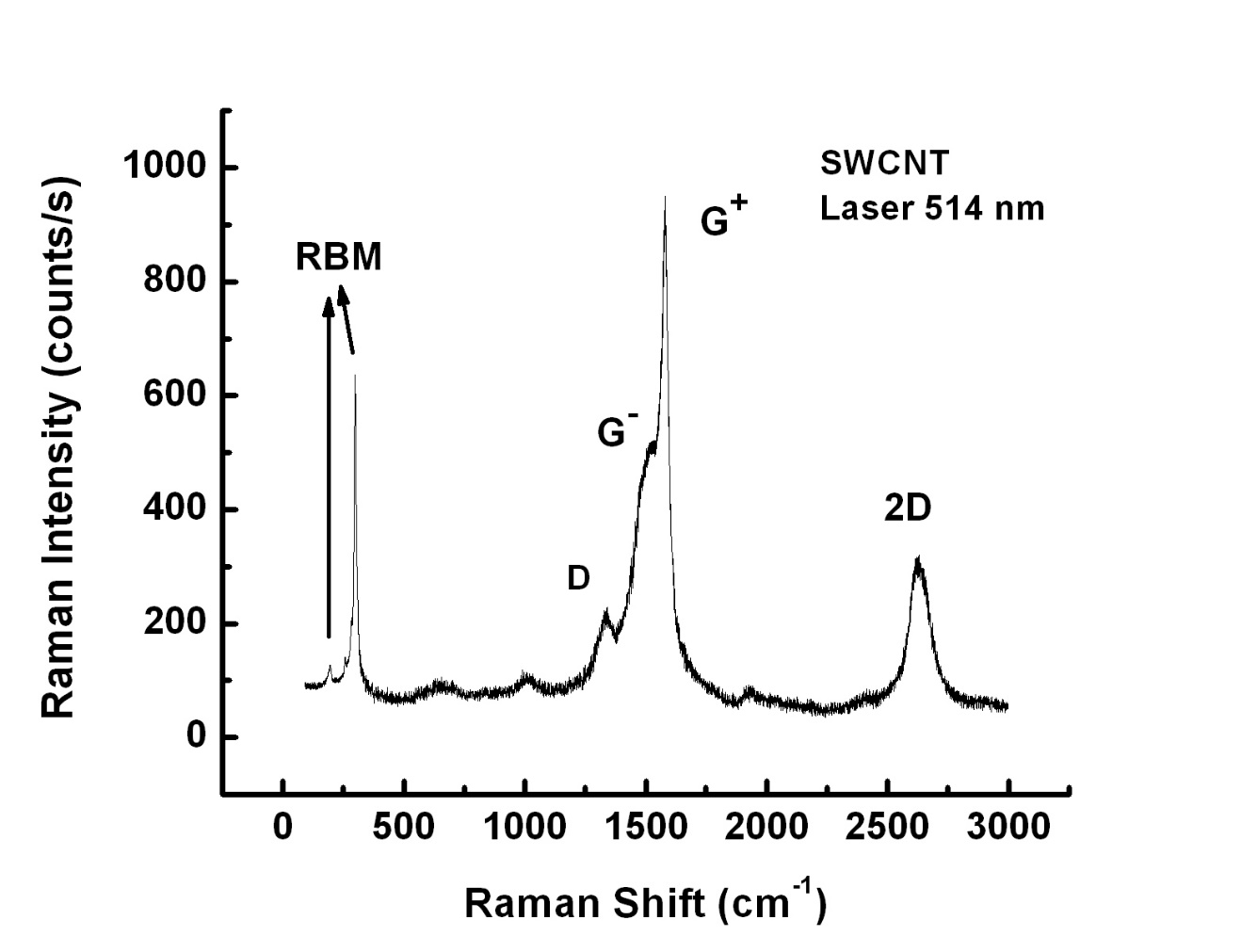


Figure S1. Raman spectrum of SWCNTs. The main features of the Raman spectra are the radial breathing mode (RBM) at 196 and 299 cm^-1^, where all of the atoms are moving in phase in the radial direction, and the G-band modes, which stem from the fundamental Raman-active mode in graphite. Also, strongly dispersive modes, denoted by the D-band at 1340 cm^-1^ and its harmonic 2D banda at 2622 cm^-1^, are observed in CNTs, and these features are also observed in graphite and sp2 carbons, along with lower intensity features, some of which are strongly dispersive and others that are weakly dispersive or nondispersive. The G band for SWCNTs carbon nanotubes is dominated by two high-intensity peaks denoted by G+ (upper frequency ωG+= 1580 cm^-1^ feature associated with carbon atom displacements along the nanotube axis) and G - (lower frequency ωG- = 1509 cm^-1^ feature associated with atomic displacements in the circumferential direction).


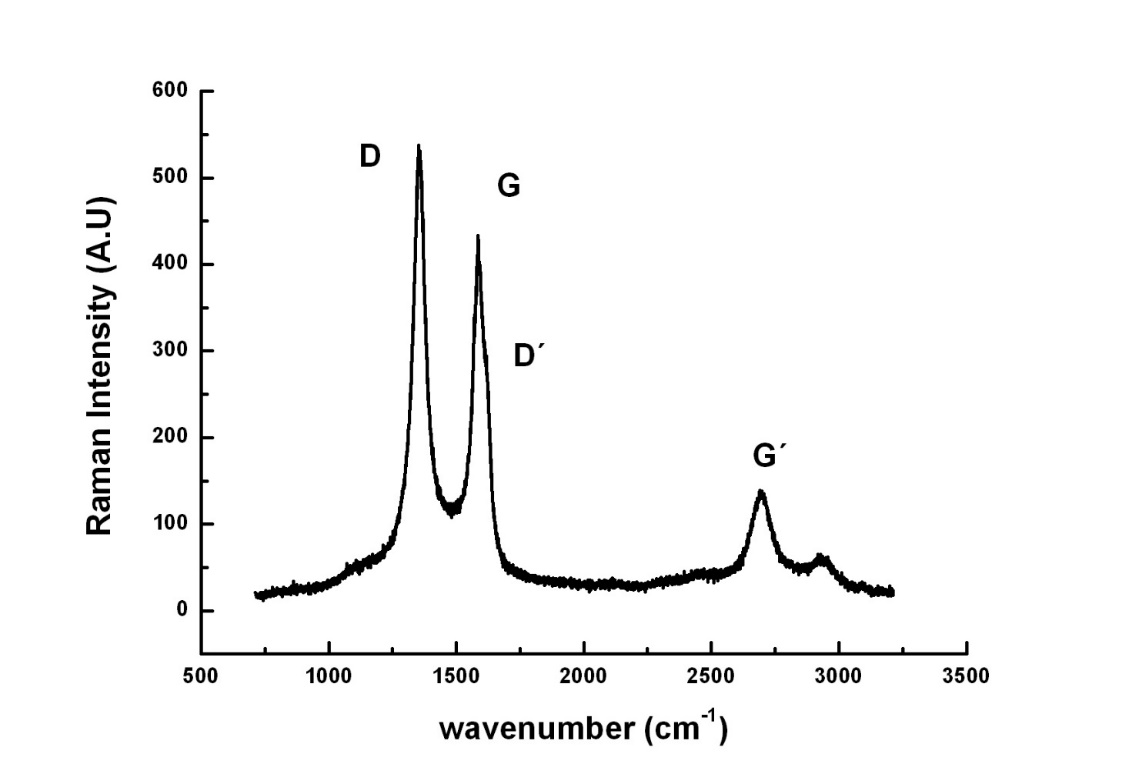

Figure S2. Raman spectrum of MWCNTs. We observe the characteristic phonons of CNTs: The dispersive disorder D band associated with defects (1355 cm^-1^), the G band (1586 cm^-1^), assigned to the in-plane vibration of the C–C bond with a shoulder around 1620 cm–1(D´band), typical of defective graphite-like materials. Like in HOPG graphite, the Raman spectrum also exhibits a band at 2692 cm^-1^ called the 2D band, a resonant two-phonon process and attributed to the overtone of the D band, which can be well fitted with a single Lorentzian peak with a 57cm^-1^ full width at half maximum (FWHM). This suggests a graphite-like behavior with weak inter-layer coupling. The low-frequency region does not show any radial breathing mode (RBM)(Not shown here). The external shells have diameters around 10nm and thereby a RBM near 25cm^-1^ out of our experimental frequency window. Besides, even if we involve small diameters for innermost shells in the1nm range, the excitation laser energy is non-resonant. This effect is in agreement with the fact that RBM modes are much weaker in MWCNT than in SWCNT owing to the intershell mechanical coupling and the broadening of the vanHove singularities(vHs).


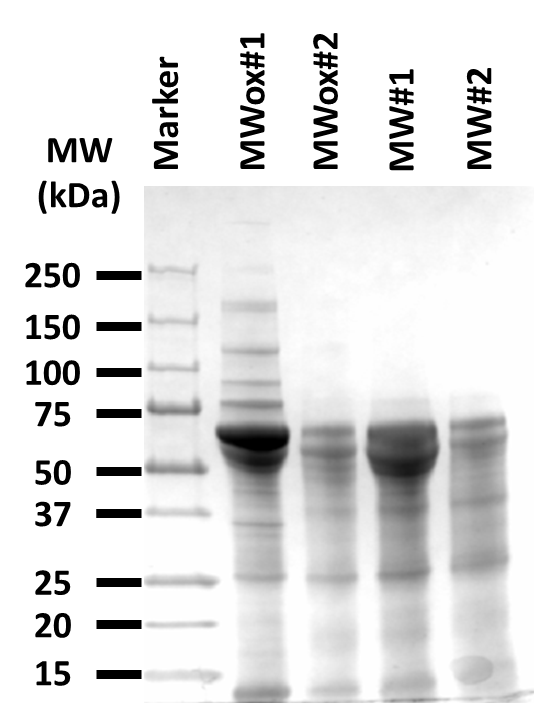


Figure S3. SDS-PAGE analysis of the bovine serum proteins obtained after the incubation with two different o-MWCNTs (MWox#1 and MWox#2) and two different pristine MWCNTs (MW#1 and MW#2).


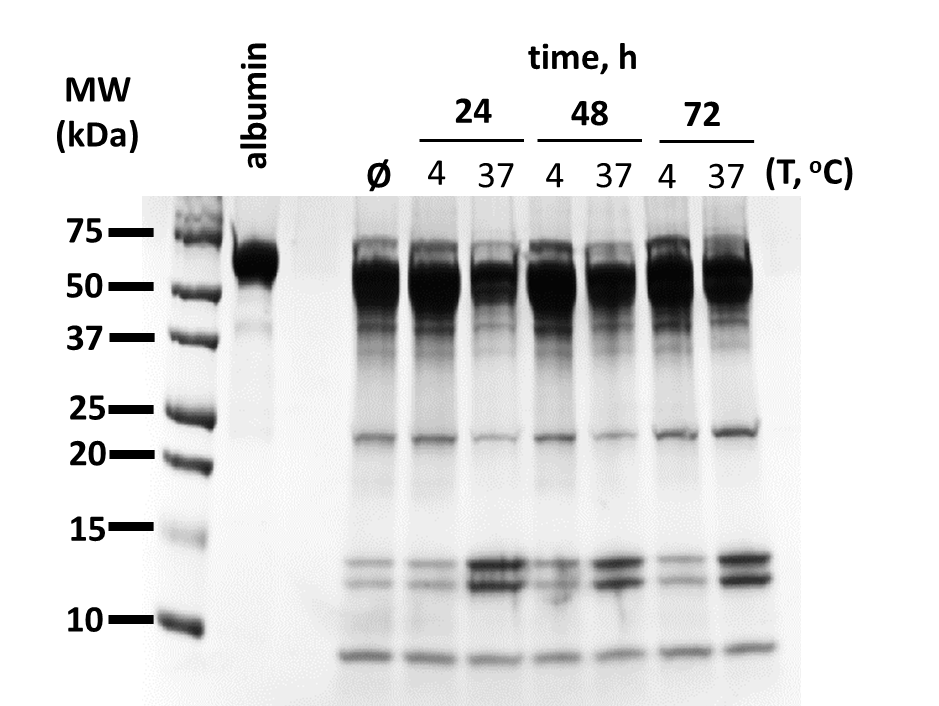


Figure S4. SDS-PAGE analysis of the protein attached to the MWCNTs´ surface after the incubation with bovine serum at 4 ºC and 37 ºC during 24, 48, and 72h.


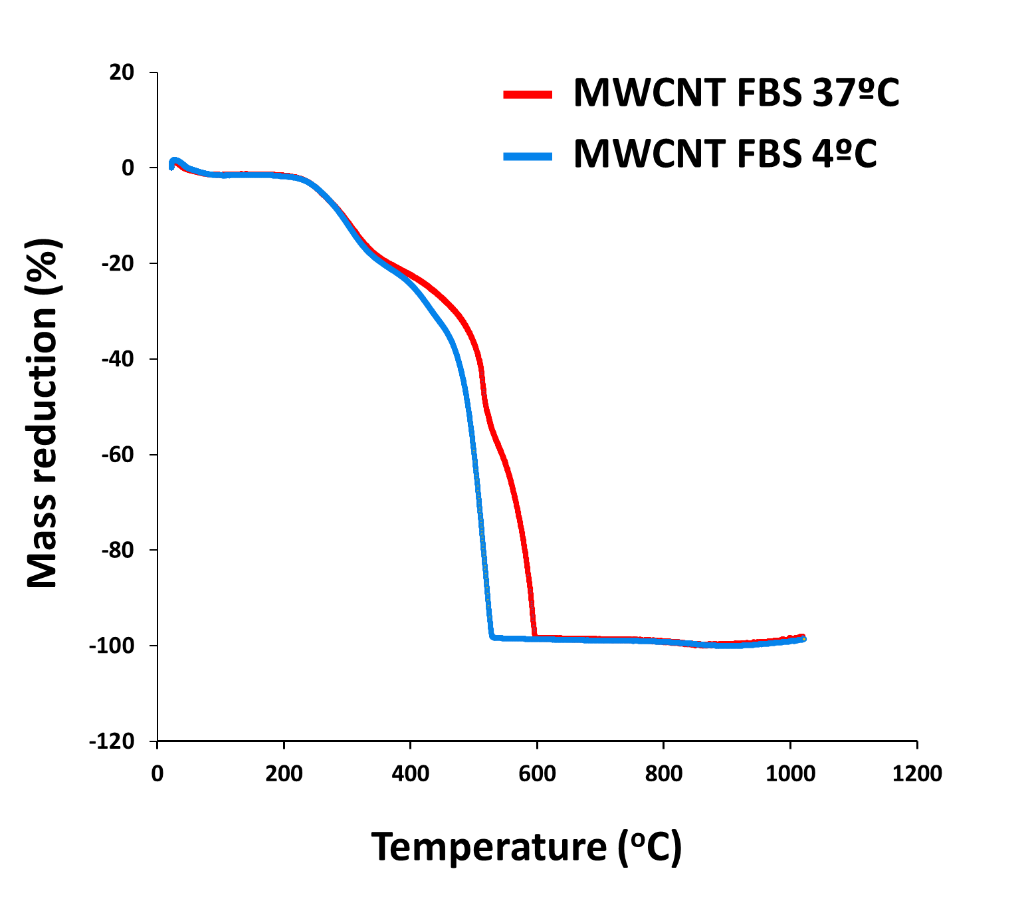


Figure S5. Thermogravimetric analysis (TGA) of MWCNTs functionalized at 4 ºC (blue line) and 37 ºC (red line) during 24 h with bovine serum.


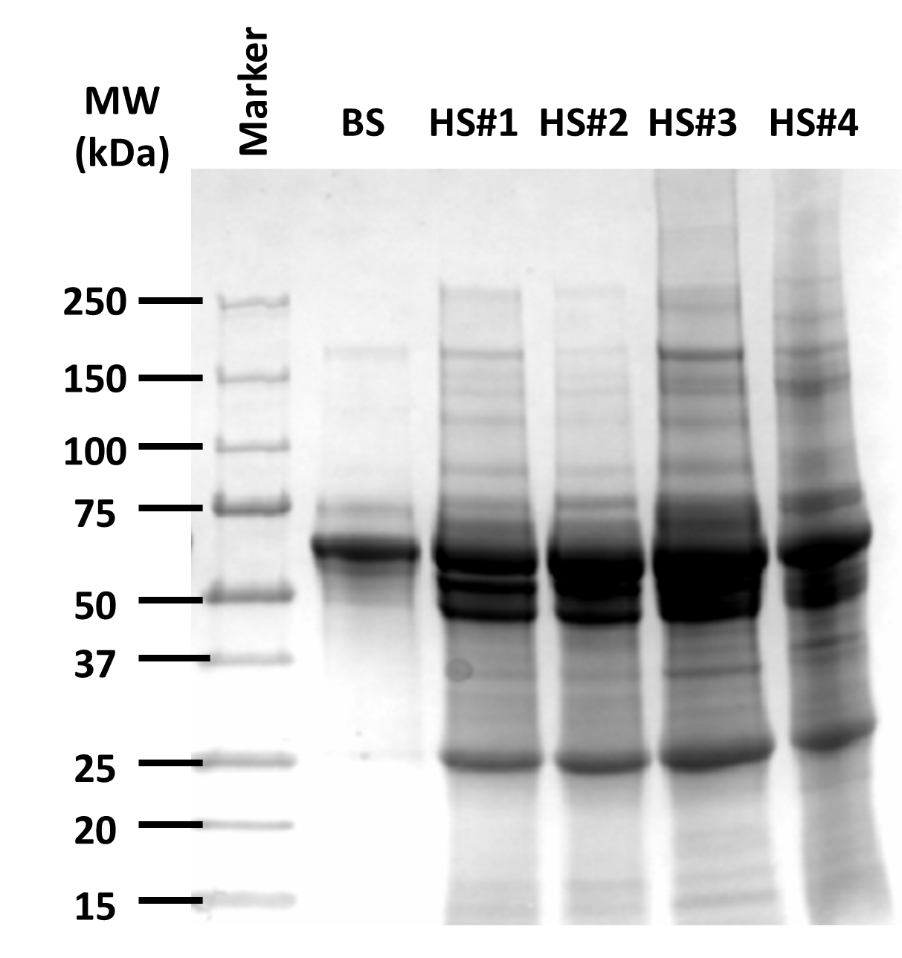


Figure S6. SDS-PAGE analysis of the protein attached to the MWCNTs´ surface upon incubation during 24h with bovine serum (BS) as control, compared to 4 different healthy human sera (HS)


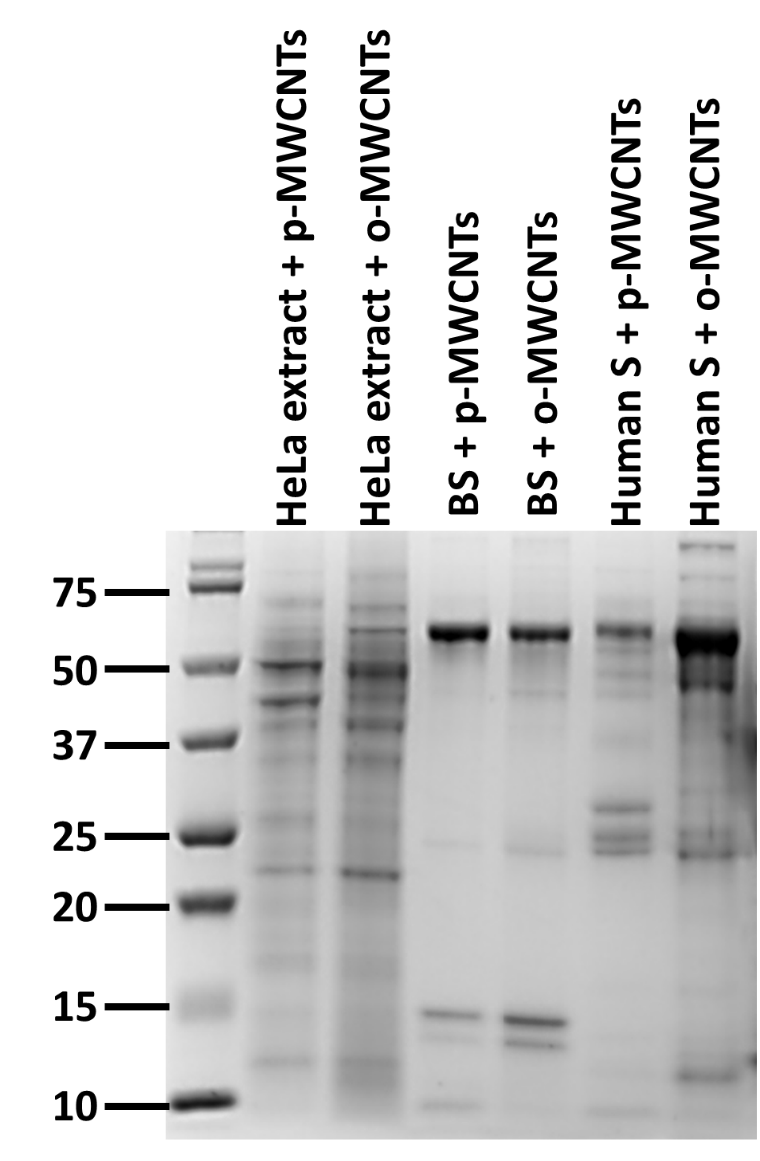


Figure S7. SDS-PAGE analysis of the protein corona obtained upon incubation of MWCNTs and o-MWCNTs with HeLa cell protein extract, bovine serum (BS), and human serum.


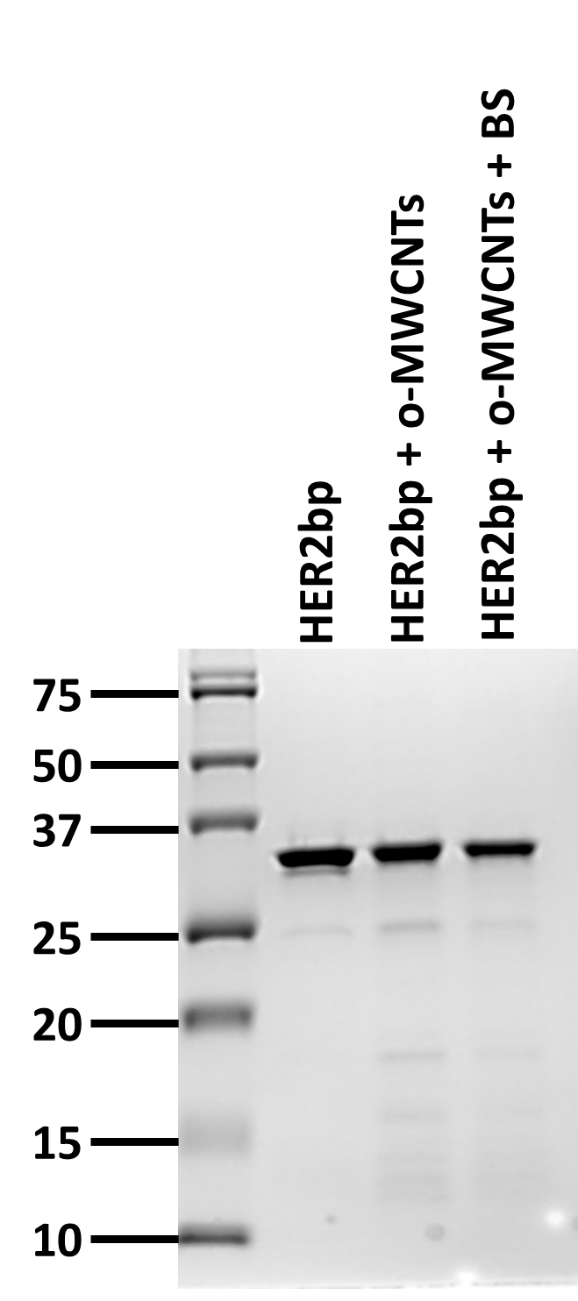


Figure S8. SDS-PAGE analysis of the purified HER2bp, oxidized MWCNT functionalized with HER2bp and the same nanotubes after 16 h incubation with bovine serum (BS).

|  | **Human Serum #1** | **Human Serum #2** | **Human Serum #3** | **Human Serum #4** |
| --- | --- | --- | --- | --- |
| *Glucose mg/dL (76-110)* | 83 | 101 | 106 | 116 |
| *Urea mg/dL (10-50)* | 43 | 51 | 56 | 18 |
| *Creatinin mg/dL (0,6-1,3)* | 1,25 | 1 | 0.71 | 0.81 |
| *Uric acid mg/dL (3,4-7)* | 5,4 | 5.3 | 3.9 | 3.5 |
| *ALT/GPT U/L (1-40)* | 21 | 18 | 20 | 17 |
| *Gamma-GT U/L (11-49)* | 45 | 33 | 20 | 6 |
| *Alkalin Phosp U/L (37-114)* | 99 | 74 | 129 | 58 |
| *Total bilirubin mg/dL (01-1)* | 0.45 | 0.31 | 0.32 | 0.24 |
| *Total cholesterol mg/dL* | 208 | 155 | 188 | 209 |
| *Triglycerides mg/dL* | 145 | 78 | 128 | 116 |
| *Calcium mg/dL (8,1-10,4)* | 10.04 | 9.59 | 9.58 | 9.17 |
| *Sodium mEq/dL (135-145)* | 146 | 149 | 147 | 148 |
| *Potasium mEq/dL (3,6-5,2)* | 3.94 | 4.24 | 3.59 | 4.07 |
| *Total protein g/dL (6-8,3)* | 7 | 6.6 | 7.6 | 6.6 |

Table S1. Biochemical parameters of the different samples of human sera

|  | **MWCNTs Nanocyl** | **MWCNTs Sigma** | **o-MWCNTs Nanocyl** | **SWCNTs Sigma** |
| --- | --- | --- | --- | --- |
| Length (μm) | 1.5 | 2.5-20 | 1.5 | 100 |
| Average Diameter (nm) | 9.5 | 6-13 | 9.5 | 3-5 |
| Purity (%) | 95 | >98 | 95 | > 98 |
| Carbon Purity (%) | > 95 | 99 | > 80 | > 98 |
| Surface Area | - | 216 m^2^/g | - | >800 m2/g |
| COOH groups (surface modification) | - | - | % > 8 | - |
| Density |  | ~2.1 g/mL at 25 °C |  | 1.7-1.9 g/cm3 at 25 °C |
| Lenght | 1.5 µm | 2.5-20 μm | 1.5 µm | 300-500 μm (in forest) |

Table S2. Characteristics of the nanotubes used.
